# Development of Flexible Medical Electrodes Using Carrageenan-Based Bioplastics with the Addition of Conductive Hybrid Materials Graphite and Silver Nanoparticles

**DOI:** 10.1021/acsomega.3c06987

**Published:** 2023-12-04

**Authors:** Fathur Rahman, Damar Rastri Adhika, Akhmad Zein Eko Mustofa, Suprijanto

**Affiliations:** † Magister of Nanoscience and Nanotechnology, Graduate School, ITB, Jl. Ganesa No. 10, Bandung 40132, Indonesia; ‡ Advanced Functional Materials Research Group, Faculty of Industrial Technology, Institut Teknologi Bandung, Jl. Ganesa No. 10, Bandung 40132, Indonesia; § Research Center for Nanoscience and Nanotechnology, ITB, Jl. Ganesa No. 10, Bandung 40132, Indonesia; ∥ Instrumentation and Control Research Group, Faculty of Industrial Technology, ITB, Jl. Ganesa No. 10, Bandung 40132, Indonesia

## Abstract

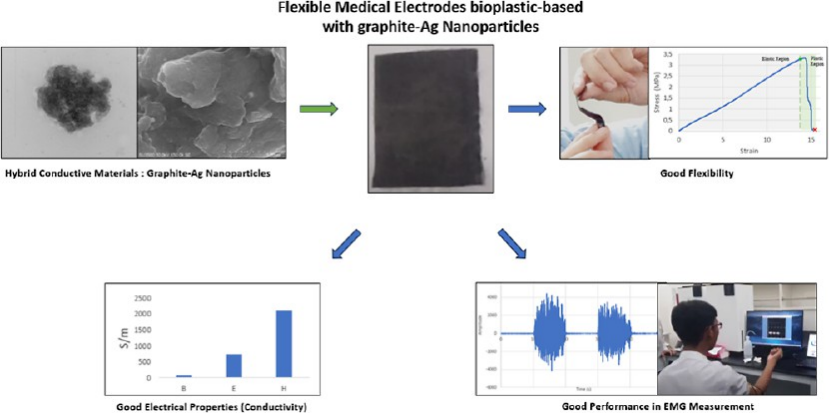

Electrodes are crucial in medical devices, specifically health monitoring devices for biopotential measurements such as electrocardiography, electromyography (EMG), and electroencephalography. The commonly used rigid electrodes have limitations in their skin-electrode contact quality since they cannot conform to the skin’s surface area and body contours. Flexible electrodes have been developed to better conform to the body’s surface contours, improving ion transfer and minimizing motion artifacts, thereby enhancing the signal-to-noise ratio (SNR). Bioplastic substrates based on carrageenan have been chosen for their safety, abundance, flexibility, and ease of customization. Hybrid materials of graphite and silver nanoparticles (graphite-AgNPs) exhibit high electron capacitance, low charge transfer resistance, and superior surface catalytic activity. These make them ideal as conductive fillers for bioplastics to achieve good electrical characteristics as electrodes. The effect of the graphite-AgNP filler concentration, graphite particle size, and flexible electrode thickness was evaluated to assess their impact on the electrical and mechanical properties of the fabricated flexible electrodes. The graphite-AgNP fillers were incorporated into a bioplastic matrix, resulting in flexible electrodes with improved conductivity with increasing percentages of graphite-AgNP at the expense of flexibility. The thickness of the flexible electrode was varied to evaluate its effect on the conductivity. A graphite size reduction was performed to improve the electrical properties while maintaining the mechanical properties. The most optimal variation of flexible electrodes with desirable electrical and mechanical properties was achieved by adding 25% graphite-AgNP to the carrageenan, using graphite particles of 400–700 nm, and using the thinnest electrode. The optimized electrode also exhibited an improved SNR value in EMG signal measurements compared to conventional Ag/AgCl electrodes. This research presents a novel approach to developing environmentally friendly, customizable, and flexible electrodes for medical applications.

## Introduction

**Figure 1:**
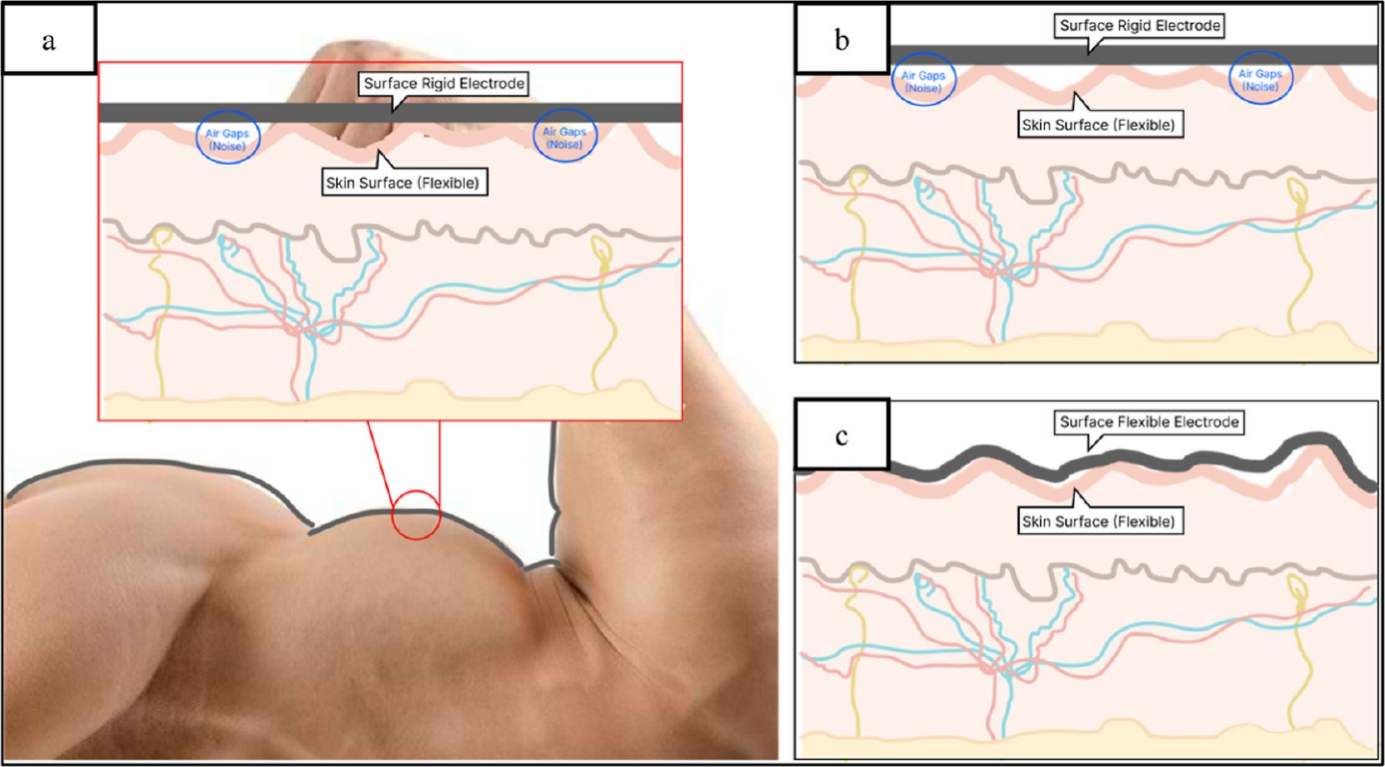
(a) Interface area electrode and skin/body contour, (b) rigid electrode, and (c) flexible electrode.

Electrodes are a sensor to measure the biopotential of the human body in the process of diagnosing or monitoring symptoms of various diseases, including electromyography (EMG), electroencephalography (EEG), and electrocardiography (ECG).^1^ These electrodes act as connectors and interfaces between the body’s internal and external electrical systems. Good contact between the electrode surface as a transducer and the skin surface plays a crucial role in the electron transfer process from negatively charged ions within the body to the electrical output of the electrode in the form of voltage.^2^ One important development is the advancement of flexible electrodes for biopotential measurement as alternatives to standard metal-based electrodes such as Ag/AgCl or gold. This development is crucial because flexible electrodes can conform to the body’s surface contours, resulting in better electrode contact and improved signal-to-noise ratio (SNR) by enhancing ion transfer from the skin surface to the electrode and minimizing motion artifacts (Figure 1). Additionally, flexible electrodes offer greater comfort during long-term use and are often required for specific purposes, such as patient monitoring.

Various flexible electrode substrates have been used in the development of electrodes, including the use of parylene C as a substrate with the addition of Ag/AgCl as a conductive material,^3^ PDMS with graphene induction using laser methods,^4^ fabric treated with TPU added with MWCNTs and Ag composites,^5^ and cotton fabric as a substrate with the addition of PANI and silver nanoparticles (AgNPs).^6,7^ These flexible electrodes could perform well to replace rigid, conventionally used Ag/AgCl electrodes based on the SNR value evaluation, as seen in Table 1 for previous studies on flexible electrodes.

**Table 1:** Research on Flexible Electrodes and Materials Development

no	materials	substrates	type	SNR (dB)	references
1	MWCNTs/PDMS composites on Ag	TPU on fabric	dry	23.1	(Masihi et al., 2022)
2	laser-induced graphene	PDMS	dry	32	(Wang et al., 2021)
3	Ag/AgCl	parylene C	dry	23.8	(Peng et al., 2013)
4	Ag/AgCl	conventional	wet	21.2	(Masihi et al., 2022)
5	AgNP PANI with gum acacia	cotton Fabric	dry	24.4	(Nurlis et al., 2022)
6	AgNP PANI with hydrazine hydrate	cotton Fabric	dry	15.8	(Nurlis et al., 2023)

In this study, the substrate for fabricating flexible electrodes is carrageenan-based bioplastic added with a conductive hybrid material of graphite powder and AgNPs (graphite-AgNPs). Carrageenan was selected because it is a safe biopolymer for prolonged direct contact with the skin^8,9^ and for its environmental friendliness.^10^ The use of the hybrid material of graphite-AgNPs as a filler in the bioplastic for flexible electrode fabrication is a good choice because it has good conductivity, an easy synthesis process, and a low price so it has the potential to be mass fabricated for conventional needs.^11^

Graphite is widely used as a filler material in polymers, plastics, and epoxy to increase conductivity.^12^ Meanwhile, one of the advantages of using AgNP is the surface area to the volume that can be adjusted for various needs.^13^ Consequently, using graphite-AgNP electrodes results in a higher current than graphite electrodes alone, thereby increasing the electrical capacitance in the hybrid material and reducing charge transfer resistance. The hybrid material also exhibits superior conductivity.^14^

The optimization of the bioplastic flexible electrodes was conducted by varying several parameters in this study, including the percentage of conductive graphite-AgNP hybrid material addition, reduction of graphite particle size, and variation in the thickness of the flexible electrodes. Physical and chemical properties characterization of flexible electrodes were performed to understand parameter variations’ effects and to find the best composition of flexible electrodes that closely approximates the characteristics of conventional electrodes for EMG measurement. This evaluation is supported by multiscale characterization, which involves material characterization at the nanoscale using a particle size analyzer (PSA), Fourier-transform infrared spectroscopy (FTIR), and transmission electron microscopy (TEM); at the microscale characterization using X-ray fluorescence (XRF) and scanning electron microscopy (SEM); as well as macroscale characterization to evaluate the electrical and mechanical properties of the electrode. The electrical properties are measured by considering conductivity, resistance, impedance, and capacitance by using the four-point probe (FPP) method and impedance analyzer. Meanwhile, the tensile testing method evaluates the mechanical properties through the tensile strength, Young’s modulus, shear modulus, and elongation percentage. The fabricated flexible electrodes’ performance during the EMG signal measurement was compared with conventional Ag/AgCl electrodes. The results indicate that the most optimum variation of the fabricated flexible electrode could outperform the performance of conventional electrodes within the EMG signal frequency range of 10–500 Hz.^15^

## Materials and Methods

### Materials

Carrageenan, graphite powder, and equates were obtained from a local market in Indonesia. HCl was purchased from Merck, Germany. NaOH, HNO_3_, H_2_SO_4_, SnCl_2_, methanol, isopropyl alcohol (IPA), ammonia solution, glucose, and poly(ethylene glycol) were obtained from a local chemical store in Indonesia. Silver nitrate (AgNO_3_) was obtained from an Indonesian state-owned mining and metals company (PT Antam).

### Synthesis of Nanosized Graphite

Nanosized graphite was obtained using the sonication method. The ultrasonication method was conducted by modifying a previous study^16,17^ by mixing graphite powder into a solution of IPA. The modification was done by improvising several ultrasonication parameters, such as graphite concentration in the solution, frequency, and duration. Ultrasonication was performed by mixing 2 g of graphite powder into 800 mL of IPA or a graphite concentration of 2.5 mg/mL in IPA. Then, the ultrasonication was performed using a 3000 MP-Biologics Lab Equipment-ultrasonic homogenizer at 60 kHz for 8 and 15 h, respectively. Each sample was centrifuged at a speed of 3000 rpm for 60 min (4× 15 min). Finally, the sample was filtered and dried.

### Carboxyl Functionalization of Nanosized Graphite

About 1 g of nanosized graphite was treated in a 200 cm^3^ solution of mixed acids consisting of HNO_3_ and H_2_SO_4_ (1:3 v/v) and refluxed at a temperature of 110 °C for 8 h to produce carboxyl-functionalized graphite nanosized with –OH and –COOH bonds. After refluxing, the graphite powder was filtered and separated from the solution using centrifugal force utilizing the Hitachi high-speed refrigerated centrifuges. Subsequently, it was washed with deionized water and dried at 95 ± 3 °C for approximately 6 h. Thus, the carboxyl-functionalized graphite nanosize was obtained.^14^ The carboxyl-functionalized graphite powder was then characterized by using FTIR to observe the formation of –OH and –COOH functional groups.

### Attachment of AgNP onto the Surface of Nanosized Graphite

The synthesis method of AgNP attachment onto the surface of graphite is based on the study by Shivakumar et al. using the electroless plating method.^14^ First, 2 g of oxidized graphite powder was treated with 50 cm^3^ of 20% SnCl_2_, stirred for 10 min, and filtered. The obtained graphite powder was then treated with 30 cm^3^ of glucose solution, 20 cm^3^ of methanol, and 1 g of poly(ethylene glycol), stirred for 10 min, filtered, and transferred to a 250 cm^3^ of silver bath solution (3 g of AgNO_3_ in 250 cm^3^ of aqua dest) along with 0.6 g NaOH, stirred for 15 min. Finally, the ammonia solution was added dropwise to get a clear solution, stirred for 45 min, filtered, and dried.^14^ The initial formation of AgNPs is based on the application of a reducer and an oxidizer on the surface of the graphite substrate (Sn^2+^ and Ag^+^), with the redox reaction process as follows^18^
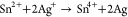
1

The glucose solution acts as an aldehyde monosaccharide that will undergo oxidation to form a carboxyl group. Ethylene glycol acts as a reducing agent for silver metal ions. At the same time, methanol functions as a solvent for the aldehyde group, which can accelerate the reaction rate and balance the reaction. The next step involves using aqua dest as a solvent, NaOH as a pH regulator to create an alkaline environment, and AgNO_3_ as the material to be reduced to obtain AgNPs. In alkaline conditions, poly(ethylene glycol) undergoes base hydrolysis to produce an aldehyde that oxidizes and releases electrons
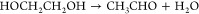
2
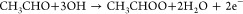
3

Simultaneously, a reaction occurs between OH^–^ ions and silver ions in the formation of silver oxide
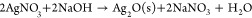
4

Subsequently, the addition of ammonia triggers a complex reaction with silver oxide and sodium nitrate, resulting in the formation of silver ammonium nitrate
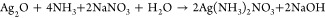
5
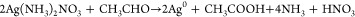
6

The release of electrons reduces silver ions to AgNPs, producing a clear, transparent solution. Similar to silver ions, the generated AgNPs also remain bound to the surface of the graphite nanosized. Finally, the AgNP layer is deposited onto the graphite surface by treating it with an alkali solution containing ammonia. As shown in eq 4, AgNO_3_, in the presence of alkali, is oxidized into silver oxide, forming Ag^+^ ions. Some of the Ag^+^ ions produced are reduced to Ag^0^, while the leftovers produce silver diamine complexes known as Tollen’s reagent upon adding ammonia. Tollen’s reagent is commonly used as a source to synthesize AgNPs as it readily undergoes reduction to Ag^0^ in the presence of aldehydes, which are then oxidized to carboxyl groups. [Ag(NH_3_)_2_]^+^, which is positively charged, can be easily absorbed onto the graphite surface through the –COOH and –OH functional groups.^19^

### Fabrication of Flexible Electrodes Using Carrageenan-Based Bioplastic with the Addition of Graphite-AgNP Hybrid Conductive Material

Carrageenan-based bioplastic was fabricated by stirring 3 g of carrageenan with 125 mL of distilled water using a magnetic stirrer for 5 min. After, montmorillonite (MMT) at a weight ratio of 20% w/w to carrageenan mass of about 0.6 g was added and stirred for 5 min at 80 °C. Then, variations of 10–25% w/w graphite-AgNP to the carrageenan mass were added to the solution while maintaining the solution temperature at 80 °C for 45 min. Afterward, glycerol at a concentration of 3% v/v of the solvent (3.75 mL) was added to the solution and stirred for 10 min. Finally, the solution was poured onto a tray measuring 22 × 26 cm and dried at 60 °C for 20 h in an oven.^8^

### Characterization of a Graphite-AgNP Hybrid Conductive Material

Physical and chemical properties were characterized at every stage of the experiment. Successful graphite functionalization was proven by FTIR (Bruker ATR) measurements that could evaluate the existing functional groups in the sample. NP characterization of size and structure was performed using the PSA NP Analyzer Horiba SZ-100 and the TEM Hitachi H9500 with an accelerating voltage of 300 kV to ensure AgNP formation. The morphology, particle distribution, and elemental composition of flexible electrodes were evaluated using a Hitachi SU3500 SEM instrument with an accelerating voltage of 10 kV and an Orbis Micro XRF elemental analyzer with an accelerating voltage of 40 kV.

### Characterization of Carrageenan-Based Flexible Electrodes

The electrical properties of flexible electrodes were tested using a digital multimeter (Keithley DMM7510), a DC source generator using the FPP method, and an impedance analyzer using Digilent’s Analog Discovery 2 pro method through conductivity, resistance, impedance, and capacitance measurements. Meanwhile, the mechanical properties of flexible electrodes were assessed by a tensile test to determine Young’s modulus, shear modulus, tensile strength, and elongation. The results of these characterizations will be utilized to evaluate their change based on variations of three parameters; the percentage of addition of the conductive hybrid material graphite-AgNP, the reduction in graphite size, and the thickness of the flexible electrode.

### Performance Test of Flexible Electrodes for EMG Signal Measurement

**Figure 2:**
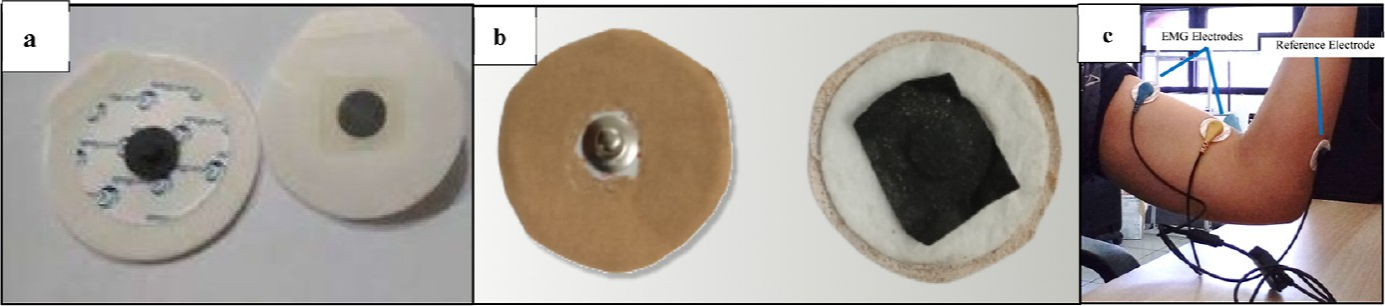
(a) EMG conventional electrodes, (b) developed flexible electrodes, and (c) EMG and reference electrodes.

The flexible electrodes were tested for the EMG signal measurement using the FlexComp Infiniti-10 channel system.^20−22^ For each EMG signal measurement, electrodes were placed at three points on the arm, as shown in Figure 2c. The first and second points were for EMG electrode placement, and the third was for reference electrode placement. The EMG signal measurements were performed four times for three variations of flexible electrodes (samples B–H) and conventional Ag/AgCl, as seen in Figure 2a,b. The measurements on the arm muscles were performed in both relaxed and contracted conditions. Each data acquisition session consisted of one cycle of relaxation and contraction, starting with a 5 s contraction, then a 5 s relaxation, then another 5 s contraction, and finally a 5 s relaxation. During the contraction phase, the arm was flexed as if lifting and gripping a weight, while during the relaxation phase, the arm was straightened without gripping anything. After obtaining the data, analysis and comparisons were made between the conventional electrode (Ag/AgCl) and the developed flexible electrodes.^23^

### Evaluation Process for the Analysis of Flexible Electrode Sample Variations

**Figure 3:**
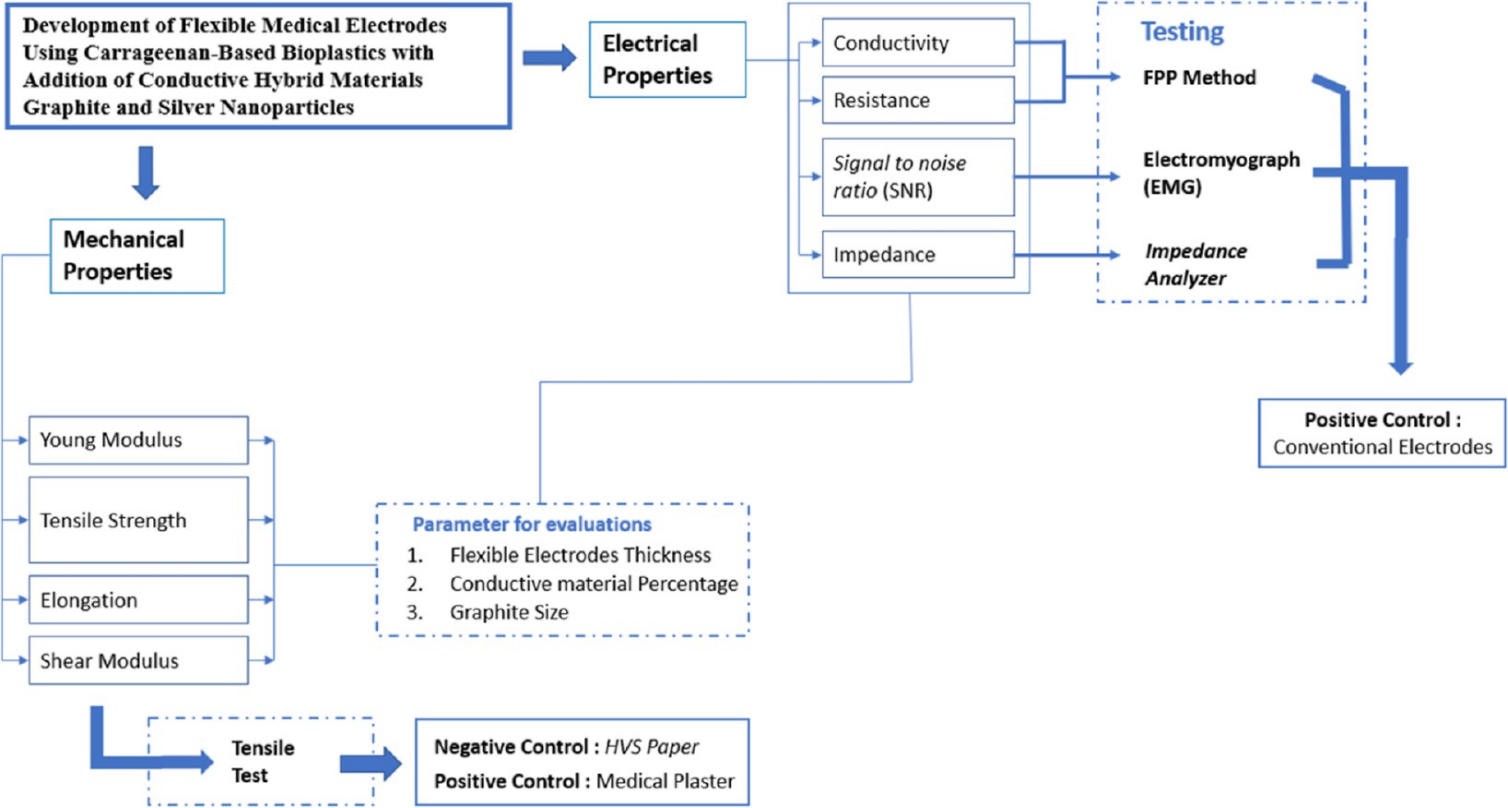
Flow diagram for the developed flexible electrodes’ characterization and evaluation.

The electrical and mechanical properties of the fabricated flexible medical electrodes were studied to evaluate their potential for measuring muscle biopotentials on the forearm for EMG devices (Figure 3). The electrode’s performance was evaluated based on three parameters of the flexible electrodes: the effect of the conductive material percentage variation (samples C, D, E, and F); the effect of flexible electrode thickness variation (samples G, H, and I); and the effect of graphite size variation (samples B, E, and H). The electrical and mechanical properties were obtained for each variation of the fabricated flexible medical electrodes. Furthermore, the flexibility assessments were performed on samples E and H to evaluate the change in flexibility along with graphite size reduction. Samples B, E, and H were chosen for the EMG measurement test because they have the same percentage of conductive materials and the same thicknesses with optimal conductivity based on the previous tests. EMG measurement results were then used to evaluate the effect of the graphite size variation on the performance of flexible electrodes. Finally, the EMG measurement results obtained with the fabricated flexible electrodes were compared to those from conventional Ag/AgCl electrodes.

## Results and Discussion

### Evaluation of Graphite-AgNP Hybrid Conductive Material

#### Ultrasonication and PSA of Nanosized Graphite

Optimizing the ultrasonication frequency, graphite concentrations, and centrifuge duration improved particle size reduction and delivered a 60.98% more efficient processing time than the reference. The particle size obtained from PSA in Table 2 shows that the average graphite size is 5061 nm after 8 h of ultrasonication. After 15 h of ultrasonication, the average size was reduced to 460 nm, indicating that ultrasonication could efficiently reduce the graphite size.

**Table 2:** Ultrasonication Parameter for Particle Size Reduction Confirmed by PSA Results

			sonication parameters			centrifuge parameters			
material	solution	graphite concentration (mg/mL)	method	freq (kHz)	dur. (h)	rpm	dur. (mins)	particle size (nm)	references
graphite								11,000	this study (initial)
graphite	IPA	3.3	ultrasonication	40	48	2000	45	1000–1100	Arlene O’nail et al.^100^
graphite	IPA	2.5	ultrasonication	60	8	3000	60	5061	this study (average)
graphite	IPA	2.5	ultrasonication	60	15	3000	60	460	this study (average)

#### Carboxyl-Functionalized Graphite Analysis Using FTIR

**Figure 4:**
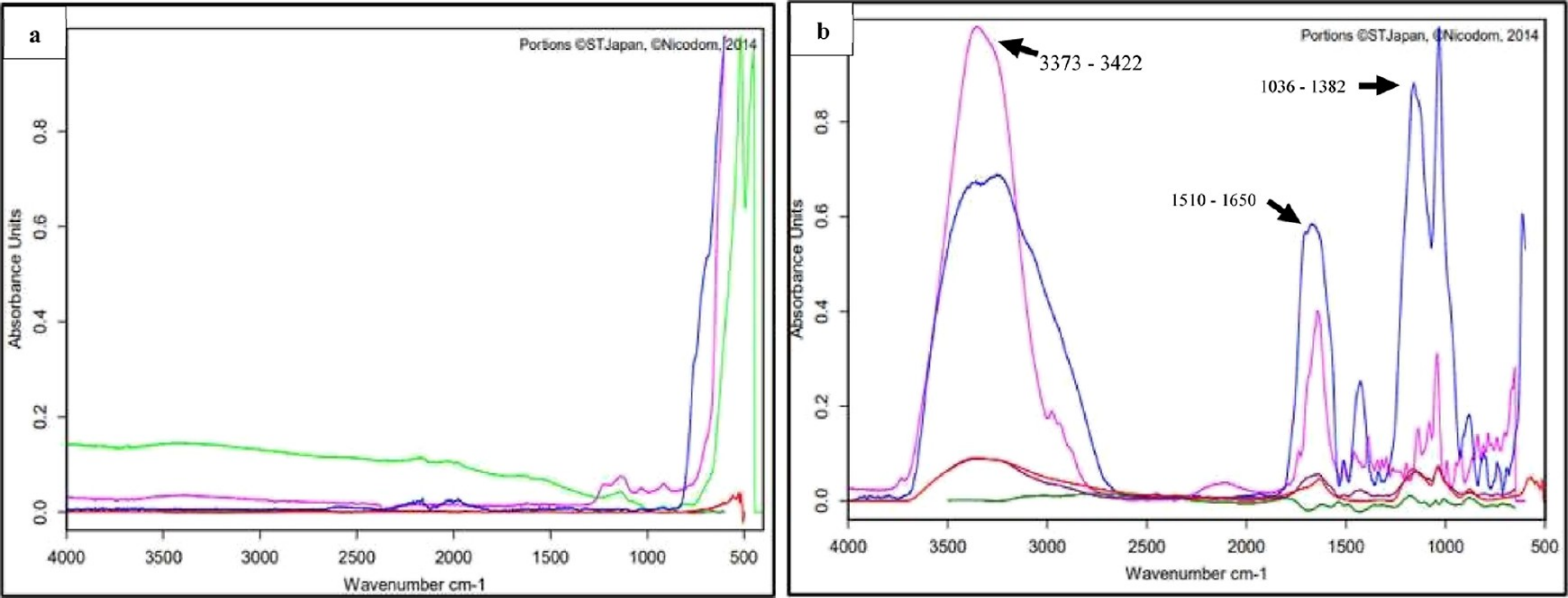
FTIR spectrum of (a) graphite and (b) functionalized graphite nanosized.

The functionalized graphite exhibits the presence of –OH and –COOH groups, as shown in Figure 4b, which differs from that of the initial graphite powder (Figure 4a). Carboxylate ion (RCOO^–^) vibration exists in the functionalized graphite FTIR spectrum (Figure 4b), in the range of 1510–1650 cm^–1^ for the asymmetrical vibration and 1280–1400 cm^–1^ for the symmetrical vibration. Carboxylate vibrations are close to the vibrations of C=O (∼1700 cm^–1^) and C–O (∼1400 cm^–1^) in the carboxylic acid form. O–H stretching vibration from carboxylic acid was observed at the 2500–3300 cm^–1^ range. The observed functional groups from the FTIR spectrum are listed in Table 3. The peaks obtained from the FTIR spectrum show that the –COOH and –OH groups have formed in the functionalized graphite. The formation of these groups is used for decorating the graphite nanosized with AgNPs.

**Table 3:** Functional Groups Observed from FTIR Analysis^24−26^

no	wave number ranges (cm^–1^)	functional group
1	1026	C–O stretching, alcohol
2	1036–1382	C–O stretching
4	1280–1400	carboxylate for the symmetrical vibration
5	1440–1395	O–H bending, carboxylic acid
6	1510–1650	carboxylate for the asymmetrical vibration
7	3550–3200	O–H stretching, alcohol
8	2500–3300	O–H stretching vibration, carboxylic acid (peak at 3000)

#### TEM Characterization of Graphite-AgNP Hybrid Material

**Figure 5:**
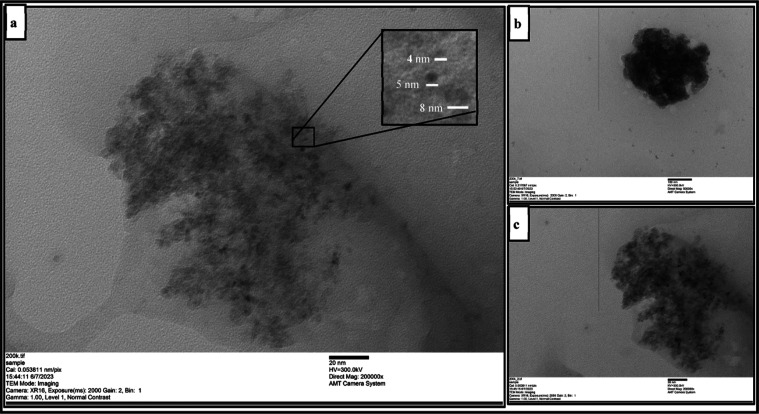
TEM images at 200k magnification of (a) particle silver in hybrid graphite-AgNP with silver particle size 4–8 nm, (b) graphite powder, and (c) bioplastic sample graphite-AgNP at 200k.

The result of imaging using a TEM is seen in Figure 5; the results show that the synthesis process of the graphite-AgNP conductive hybrid material was successfully carried out using sonicated graphite for 8 and 15 h. Black spherical particles are identified as AgNPs with particle ranges of 4–8 nm. Graphite could be seen as a darker area around AgNP in Figure 5c since graphite is thicker than the TEM grid carbon layer substrate. TEM observation indicates that the fabrication method has been optimized and successfully carried out for different graphite sizes. Figure 5 shows that the synthesis process of the graphite-AgNP conductive hybrid material was successfully carried out using sonicated graphite for 8 and 15 h. TEM image acquisition uses a carbon background so that graphite cannot be seen clearly. However, several gray areas were identified as graphite due to their darker contrast than the background. Black spherical particles above those gray areas are identified as AgNPs. These TEM results show that the method adapted from previous research has been optimized and that this method can be applied to different graphite sizes.

### Evaluation of Flexible Electrode Sample Variations

#### Variation of Flexible Electrodes Samples

**Figure 6:**
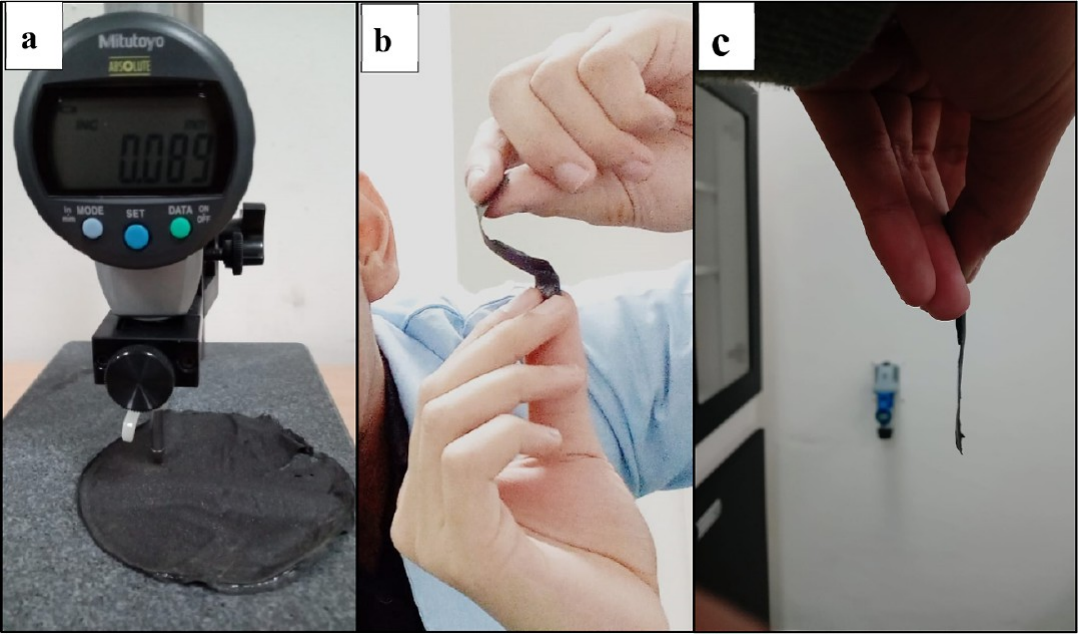
Flexible electrodes, (a) thickness measurement, (b) flexibility’s demonstration, and (c) visible side for the sample’s thickness.

Flexible electrodes were fabricated using carrageenan-based bioplastic with graphite-AgNP hybrid conductive material, as described previously in this study. Nine bioplastic-based flexible electrode variations were fabricated in this study with different types and concentrations of conductive material and several different thicknesses to find the optimum fabrication parameter to obtain a reliable bioplastic-based flexible electrode for biopotential measurement. The variation of bioplastic-based flexible electrodes is presented in Table 4 and the visualization is seen in Figure 6. The selection of sample variations was carried out, ranging from 10 to 30%. This was done because a filler composition of less than 10% may result in poor conductivity, while exceeding 30% could render the fabricated bioplastic brittle and prone to breakage.

**Table 4:** Variation of Flexible Electrodes Bioplastic with Hybrid Material Graphite-AgNP Addition

sample	composition	length (cm)	width (cm)	thickness (cm)	graphite particle size (nm)
A	carrageenan + 15% graphite	2.00	2.00	0.00139	5061
B	carrageenan + 25% graphite	2.10	2.80	0.00165	5061
C	carrageenan + 10% graphite-AgNP	2.10	2.00	0.00025	5061
D	carrageenan + 15% graphite-AgNP	2.00	2.00	0.00023	5061
E	carrageenan + 25% graphite-AgNP	2.00	2.50	0.00026	5061
F	carrageenan + 30% graphite-AgNP	2.00	2.50	0.00085	5061
G	carrageenan + 25% graphite nanosized-AgNP (*t* = 0.00087 cm)	2.00	2.50	0.00089	460
H	carrageenan + 25% graphite nanosized-AgNP (*t* = 0.00027 cm)	2.00	2.50	0.00027	460
I	carrageenan + 25% graphite nanosized-AgNP (*t* = 0.00047 cm)	2.00	2.50	0.00047	460

Figure 6 shows flexible electrodes with a visible side for thickness; the existence of limitations in the printing method causes the thickness to have a difference of around 0.02–0.04 mm in some areas, with the average thickness value given in Table 4. Electrodes with various thicknesses are fabricated using the same formula so that the parameters other than the thickness of the electrodes have the same value.

#### Effect of the Percentage of Graphite-AgNP Addition

The samples that we evaluated to observe the effect of graphite-AgNP material addition percentage on electrical and mechanical properties are samples A, B, C, D, E, and F. The evaluation results are summarized in Table 5 with 15 and 25% graphite to 10, 15, 25, and 30% addition of graphite-AgNP.

**Table 5:** Results of Measuring the Electrical and Mechanical Properties Effect of Graphite-AgNP Material Addition Percentage

sample	A	B	C	D	E	F
composition	15% graphite	25% graphite	10% graphite-AgNP	15% graphite-AgNP	25% graphite-AgNP	30% graphite-AgNP
resistance (Ω)	699.45	68.57	156.87	391.22	52.54	37.23
conductivity (S/m)	10.28	88.38	254.98	111.13	731.90	315.93
tensile strength (MPa)			5.61	5.43	4.94	4.01
elongation (%)			29.14	22.09	13.33	9.45
Young’s Modulus (MPa)			18.34	21.46	35.03	41.67
shear’s Modulus (kPa)			0.60	0.40	0.30	0.20

**Figure 7:**
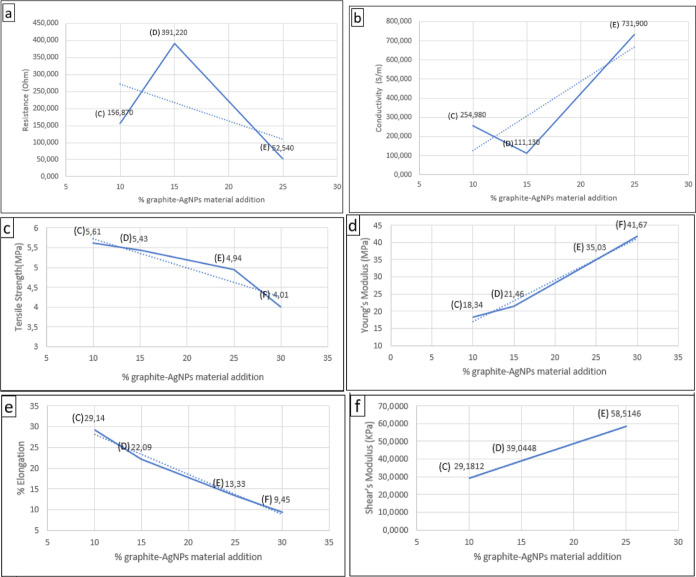
Relation of % graphite-AgNP hybrid material addition with (a) resistance, (b) conductivity, (c) tensile strength, (d) Young’s modulus, (e) % elongation, and (f) shear’s modulus.

Based on the conductivity values in Table 5, it was found that samples E and F have the highest conductivity values of 731.90 and 315.93 S/m, respectively. Meanwhile, sample F has a lower tensile strength and elongation value than sample E, so sample F was more brittle and delicate than sample E. Therefore, sample E was considered the best flexible electrode despite sample F having the highest content of the graphite-AgNP hybrid material. Based on Figure 7a, it was observed that the addition of the conductive hybrid material graphite-AgNP tended to decrease the resistance value. In contrast, in the curve in Figure 7b, it was found that increasing the percentage of graphite-AgNP also increased the conductivity. From these data, it can be informed that the best electrode based on the composition of the addition of the conductive hybrid material graphite-AgNP is sample E with a composition of 25% graphite-AgNP.

Based on the tensile strength curve (Figure 7c), it was found that the highest tensile strength was exhibited by sample C, which had the lowest composition of added hybrid material. The elongation percentage decreased as the percentage of conductive material graphite-AgNP increased, indicating that adding conductive hybrid material made the electrode less flexible. Meanwhile, Young’s modulus increases with the added conductive hybrid material percentage. Therefore, sample C, with a tensile strength of 5.61 MPa and an elongation percentage of 29.14%, was identified as the best tensile strength and flexibility combination.

#### Effect of Flexible Electrode Thickness

**Figure 8:**
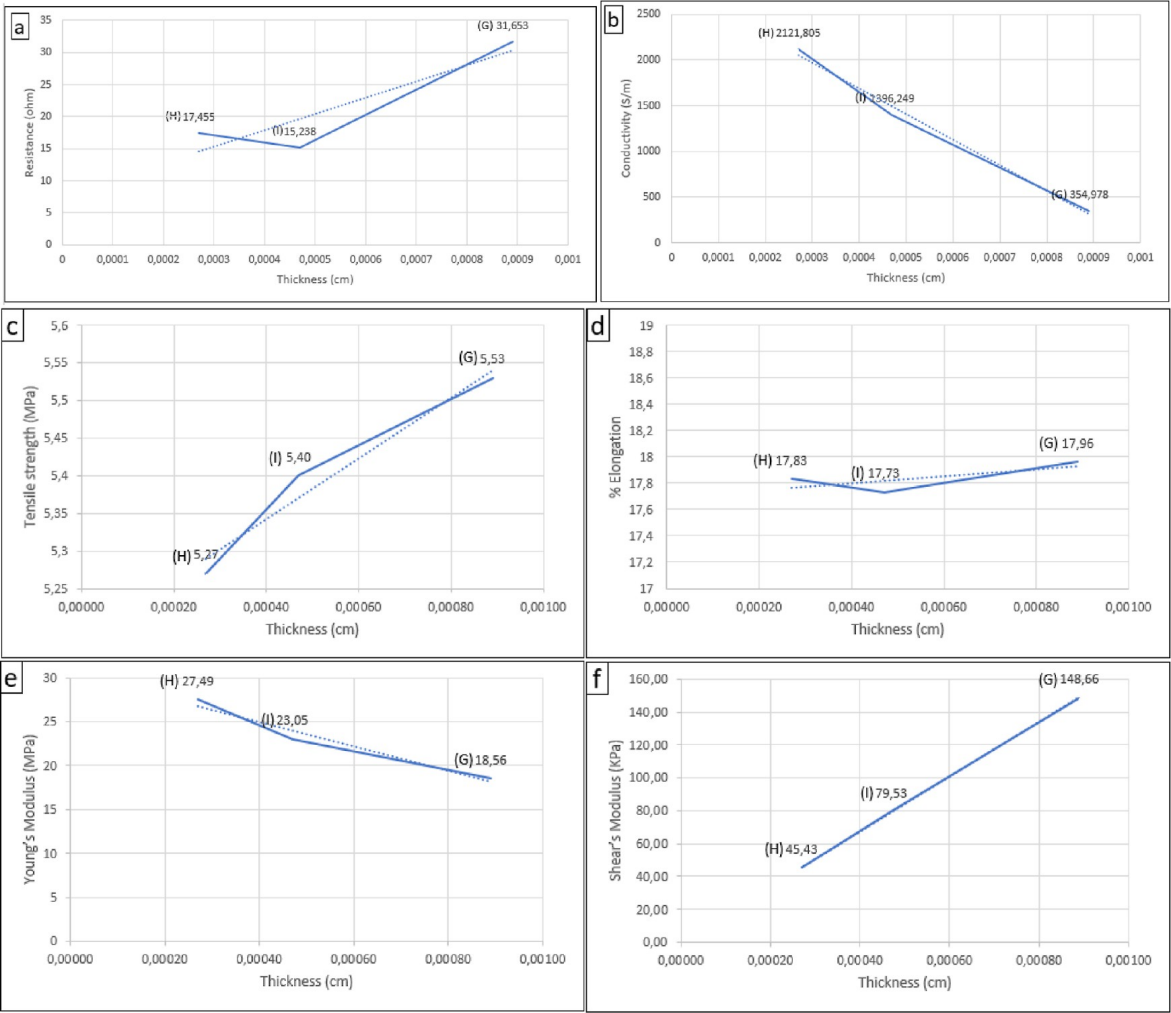
Curves of thickness flexible electrode influences to (a) resistance, (b) conductivity, (c) tensile strength, (d) Young’s modulus, (e) % elongation, and (f) shear’s modulus.

Samples G, H, and I, as samples with the same graphite particle size and composition, were evaluated to observe the effect of flexible electrode thickness variations on electrical and mechanical properties. The evaluation results are summarized in Figure 8 and Table 6. The highest conductivity value obtained was from the flexible bioplastic electrode sample H, with a conductivity value of 2121.80 S/m. In comparison, the lowest conductivity was obtained from sample G, with a value of 354.97 S/m. The highest resistance value was obtained from sample G. This indicates that the thinnest sample has the best conductivity value. For mechanical properties, sample G has the highest tensile strength, with a value of 5.53 MPa and an elongation percentage of 17.96%. The difference in elongation percentage between samples with varying thicknesses is not significantly high, indicating that thickness can increase tensile strength while maintaining elongation percentage. Sample H, as the sample with the thinnest thickness, demonstrates that the electrode becomes more flexible as the electrode thickness increases. The shear modulus increases along with electrode thickness, as observed by comparing the shear modulus values of samples G and H, which are 148.66 and 45.43 kPa, respectively. The electrical and mechanical property trends from the research findings are summarized in Table 6.

**Table 6:** Results of Measuring the Electrical and Mechanical Properties Effect of Flexible Electrode Thickness

sample	G	H	I
thickness (cm)	0.00089	0.00027	0.00047
resistance (Ω)	31.653	17.455	15.238
conductivity (S/m)	354.978	2121.805	1396.249
tensile strength (MPa)	5.53	5.27	5.4
elongation (%)	17.96	17.83	17.73
Young’s modulus (MPa)	18.56	27.49	23.05
shear’s modulus (kPa)	148.66	45.43	79.53

#### Effect of Graphite Size Reduction

Samples B–H were evaluated to observe the effect of graphite size reduction on electrical and mechanical properties, as these samples had the same addition of conductive material, which was 25% in their composition. Furthermore, in the evaluation of the percentage of added conductive material, the composition of 25% exhibited the best electrical and mechanical properties. The evaluation results are summarized in Table 7.

**Table 7:** Results of Measuring the Electrical and Mechanical Properties Effect of Graphite Size Reduction

sample	B	E	H
composition	25% graphite	25% graphite-AgNP	25% graphite nanosized-AgNP
graphite particle size (nm)	4500–6500	4500–6500	400–700
resistance (Ω)	68.57	52.54	17.45
conductivity (S/m)	88.38	731.90	2121.80
tensile strength (MPa)		4.94	5.27
elongation (%)		13.33	17.83
Young’s modulus (MPa)		35.03	27.49
shear’s modulus (kPa)		58.51	45.43
flexibility (N^–1^ m^–2^)		7.79617 × 10^17^	8.87105 × 10^17^

From Table 7, we obtained the highest conductivity value from sample H, while the lowest was from sample B. The difference between samples B and H is quite significant, with conductivity values of 88.38 and 2121.80 S/m, respectively. Sample H also has the lowest resistance value compared with the other samples. Mechanical property results in Table 7 show that reducing the graphite size can enhance the bioplastic electrode’s mechanical properties. The tensile strength value of sample H is higher than that of sample E, with values of 5.27 and 4.94 MPa, respectively. Sample H also exhibits a higher elongation percentage and flexibility compared with sample E.

Based on the elemental analysis conducted using XRF, as presented in Table 8 above, it was found that the weight percentage (wt %) and atomic percentage (at. %) of Ag and Sn in sample H were higher compared to that in sample E. These results indicate that reducing the size of the graphite material leads to an increase in the wt % and at. % content of Ag and Sn.

**Table 8:** Elemental Composition of Flexible Electrode Samples Obtained Using XRF

sample		B	E	H
graphite particle size		4500–6500 nm	4500–6500 nm	400–700 nm
wt %	Si K	29.67	9.38	9.41
	S K	32.02	3.58	1.43
	Cl K	16.17	21.94	8.09
	Ag L		44.97	60.79
	K K		18.08	10.23
	Sn L		2.05	10.04
	Si K	39.06	17.03	22.07
	S K	36.93	5.70	2.94
	Cl K	16.87	31.56	15.04
	Ag L		21.26	37.13
	K K		23.58	17.24
at. %	Sn L		0.88	5.57

**Figure 9:**
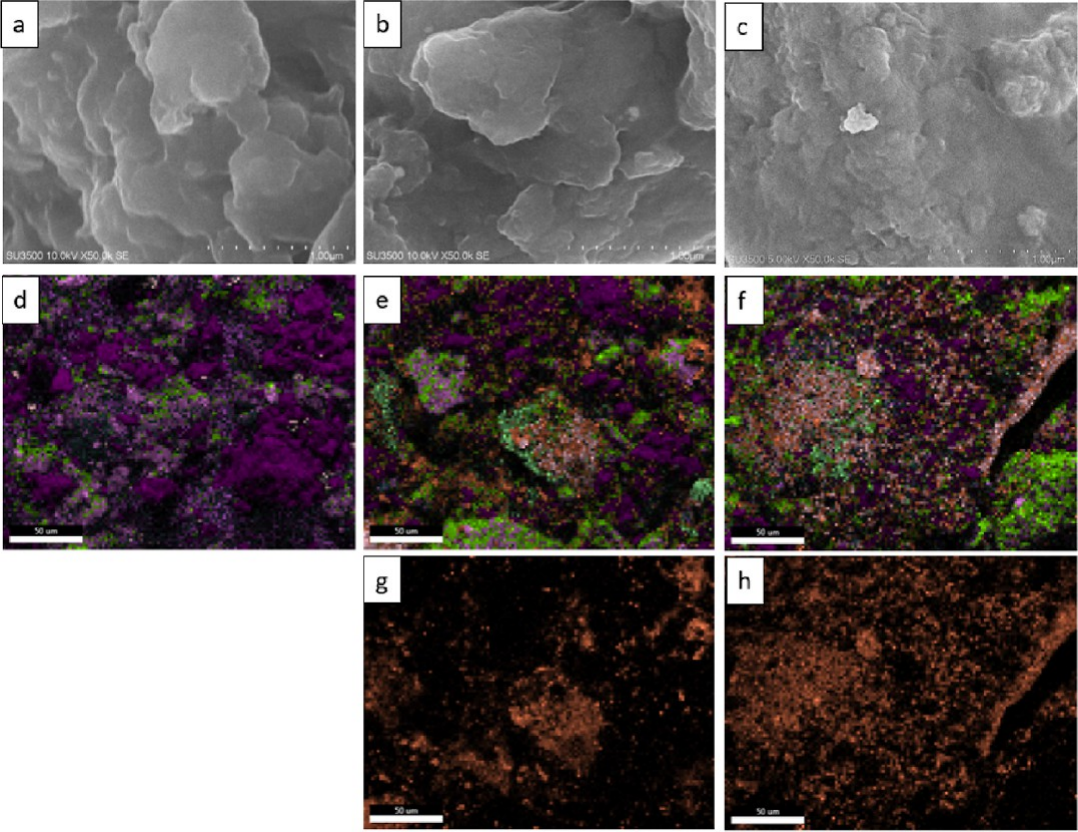
SEM imaging at 50k magnification of (a) sample B, (b) sample E, and (c) sample H; EDS mapping of (d) sample B, (e) sample E, and (f) sample F; and EDS mapping for Ag-distribution of (g) sample E and (h) sample H.

The flexible electrode samples were analyzed further using the SEM–EDS to evaluate the distribution of the conductive filler, where the results are presented in Figure 9 and Table 9. The EDS mapping for Ag distribution in Figure 9g,h shows that Ag distribution in the sample with nanosized graphite exhibits a more uniform distribution compared to that of larger-size graphite. Furthermore, as graphite size decreases, the wt % of Sn and Ag increases, as seen in Table 9. The increase in Sn and Ag elements is the consequence of graphite size reduction, which leads to an increased surface area of the graphite that promotes ion reactions between Sn and Ag on the surface; therefore, more AgNP is formed on the graphite surface. SEM–EDS results indicate that a smaller graphite size could increase AgNP formation and improve conductive filler distribution in the flexible electrode samples.

**Table 9:** Elemental Analysis Using EDS SEM of Flexible Electrodes

	wt % of element					
sample	C	Si	Ag	Sn	S	Cl
B	68.08	6.73			1.95	0.69
E	44.35	3.09	10.70	1.17	1.25	2.39
H	43.21	3.85	15.72	2.42	0.92	1.78

#### Flexibility Analysis

The flexibility values could be analyzed from the stress–strain curve obtained via the tensile test. After acquiring the preceding Young’s modulus values, the flexibility values were calculated by considering the moment of inertia of the sample, employing a simple theoretical approach described in a paper studying the flexibility of wet cellulose fibers utilizing atomic force microscope (AFM) measurement.^27^ The flexibility (*F*) of an in-plane bending of a homogeneous, symmetrical, and linear elastic cross-section is described by the conventional Bernoulli/Euler beam theory
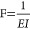
7where *E* is the modulus of elasticity and *I* is the moment of inertia of the beam structure since the thin film was evaluated in this study. The moment of inertia for a beam with a rectangular cross-section with the width (*b*) and height (*h*) given by
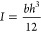
8

The moment of inertia is very sensitive to the geometrical dimensions of the sample. The flexibility of the flexible electrode sample can be calculated from eq 8 when the sample’s geometrical dimensions and modulus of elasticity are known. In this study, modulus elasticity was obtained from the tensile test, and the geometry was already known, where height (*h*) is the thickness of the samples. The results of the flexibility calculation are shown in Table 10. Bioplastic-based flexible electrodes have a high flexibility value. The flexibility value increased with the decrease of conductive fillers and sample thickness.

**Table 10:** Flexibility Values of Flexible Electrodes

sample	composition	flexibility (N^–^^1^ m^–^^2^)
C	carrageenan + 10% graphite-AgNP	2.09378 × 10^18^
D	carrageenan + 15% graphite-AgNP	2.29794 × 10^18^
E	carrageenan + 25% graphite-AgNP	7.79617 × 10^17^
F	carrageenan + 30% graphite-AgNP	1.87569 × 10^16^
G	carrageenan + 25% graphite nanosized-AgNP (*t* = 0.00087 cm)	3.66854 × 10^16^
H	carrageenan + 25% graphite nanosized-AgNP (*t* = 0.00027 cm)	8.87105 × 10^17^
I	carrageenan + 25% graphite nanosized-AgNP (*t* = 0.00047 cm)	3.08012 × 10^17^

#### Stress–Strain Curve Analysis

**Figure 10:**
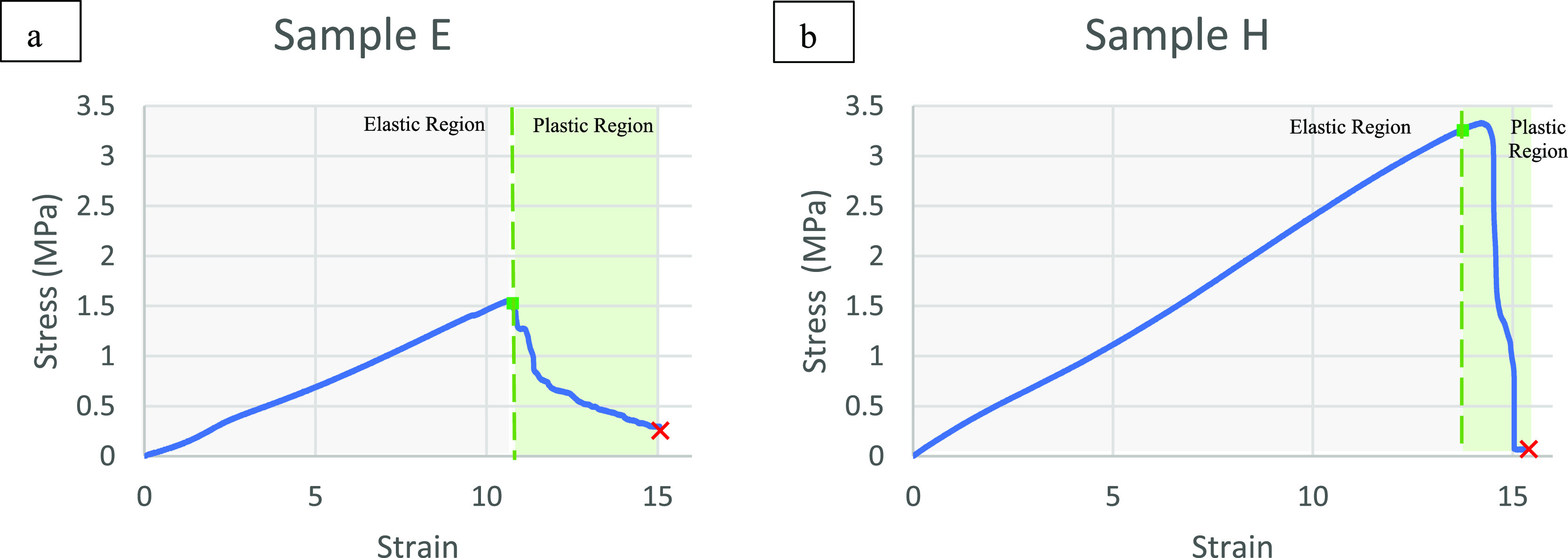
Stress–strain curve for (a) sample E and (b) sample H.

The mechanical characteristics of the bioplastic have been analyzed using the tensile test method. Samples E and H are selected for evaluation since they have very similar thicknesses and the same concentration of conductive fillers. However, sample E used unprocessed graphite, while sample H used nanosized graphite. By observation of the trends in the stress–strain curve in Figure 10, the effect of nanosized graphite on the mechanical characteristics of the fabricated flexible electrode could be determined.

The dashed green line in Figure 10 separates the phases of the elastic and plastic regions in the stress–strain curve, where the linear slope indicates the elastic region followed by the plastic region. It can also be observed that sample H, which has a smaller nanosized graphite, has a larger elastic region than sample E. However, sample H has a smaller plastic region than sample E’s plastic properties. Therefore, the strain at the breaking point indicated by a red cross on the stress–strain curve was observed at 15.04 for sample H and 14.76 for sample E, which was not significantly different for both samples. Stress–strain curve analysis indicates that reducing the graphite size to the nanoscale can enhance the mechanical properties of the bioplastic-based flexible electrodes, where the elastic region for sample H is larger than sample E.

#### Impedance Spectroscopy

**Figure 11:**
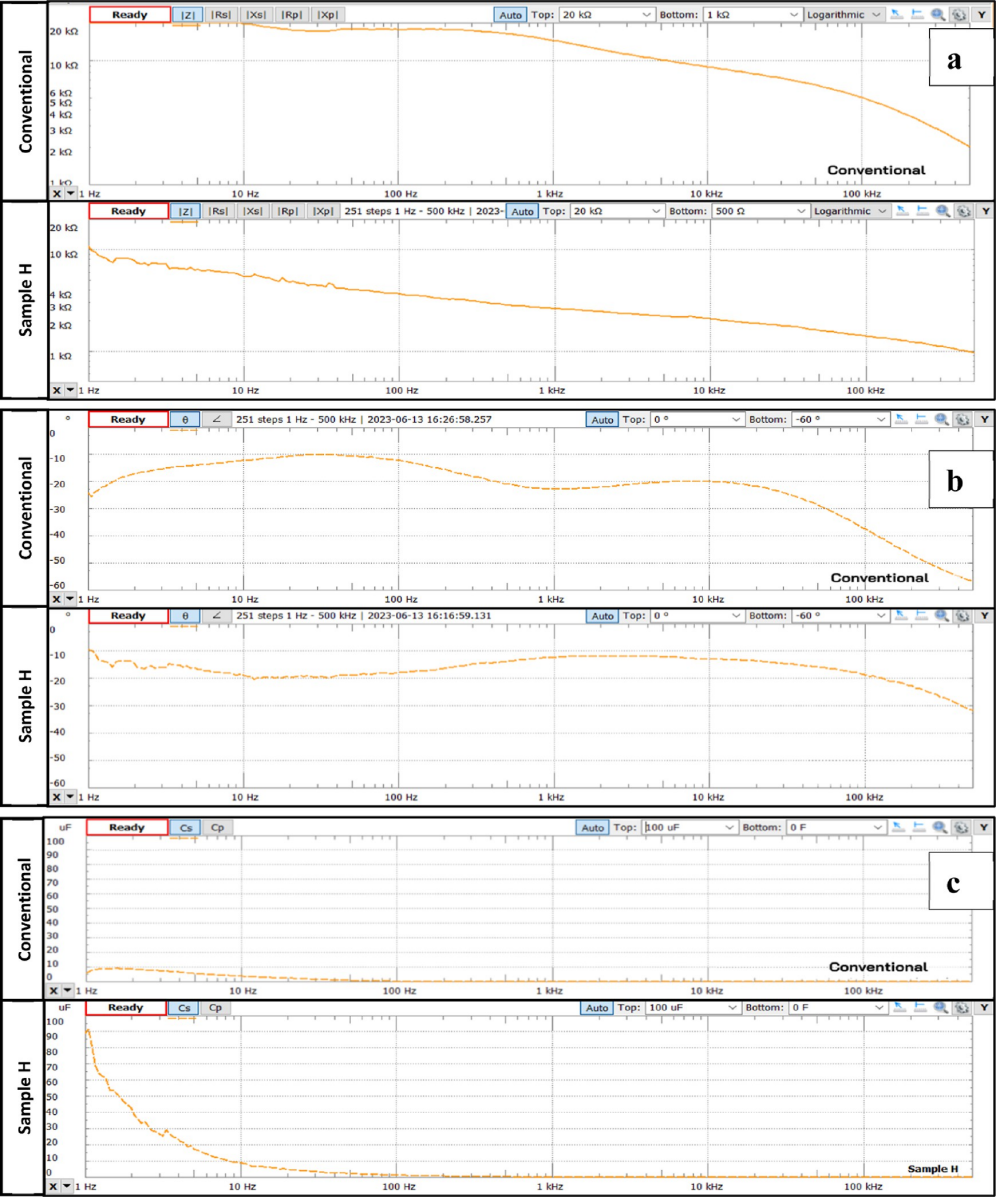
Impedance spectroscopy: (a) impedance values as a function of frequency, (b) phase values as a function of frequency, and (c) capacitance values as a function of frequency; for sample H and conventional electrode.

The impedance data were collected using samples B–H to study the effect of graphite material size. Further, samples G, H, and I were used to investigate the effect of electrode thickness. These samples were compared with conventional electrodes using an impedance analyzer within the frequency range of EMG signals, 0–500 Hz. The impedance measurement was performed, and the results are summarized in Table 11 and Figure 11.

**Table 11:** Result of Impendace Measurement

sample	impedance (Ω)
B	57328.90
E	14211.24
G	61671.51
H	6442.74
I	25911.51
conventional	26217.90

From impedance spectroscopy (Figure 11a), it can be observed that the fabricated electrodes exhibit a reactive response at low frequencies. Compared with conventional electrodes, the fabricated flexible electrodes have lower impedance values. From impedance spectroscopy (Figure 11b), it can be seen that the phase angle range for the fabricated medical flexible electrode sample H is between −8 and −35°, indicating that the fabricated electrodes exhibit capacitive characteristics similar to conventional electrodes. From capacitance spectroscopy (Figure 11c), it is known that flexible electrode sample H has higher capacitance than conventional electrodes within the frequency range of 1–100 Hz. However, for frequencies above 10 Hz, the capacitance value of the H sample is comparable to that of conventional electrodes so that the biopotential sensing performance of the H sample can be close to that of conventional electrodes in this frequency range.

**Figure 12:**
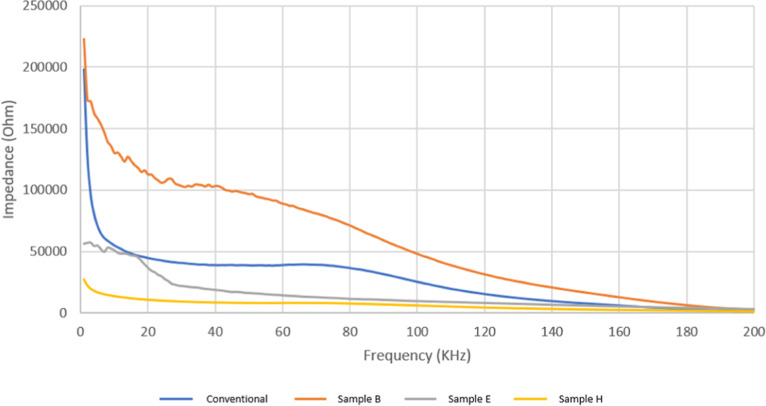
Impedance measurement of fabricated electrodes and conventional.

Sample H has the lowest impedance value of 6442.74 Ω, while sample G has the highest impedance value of 61671.51 Ω. Thus, it is found that the graphite material’s size and the flexible electrode’s thickness influence electrical impedance. Figure 12 shows that a smaller graphite material and thinner electrode thickness lead to lower impedance values. Furthermore, it can be observed that within the 0–500 Hz frequency range, the flexible bioplastic electrodes of samples E, H, and I have lower impedance values compared to conventional EMG electrodes, thus improving the electrical properties’ quality of the flexible electrode.

#### EMG Measurement

**Figure 13:**
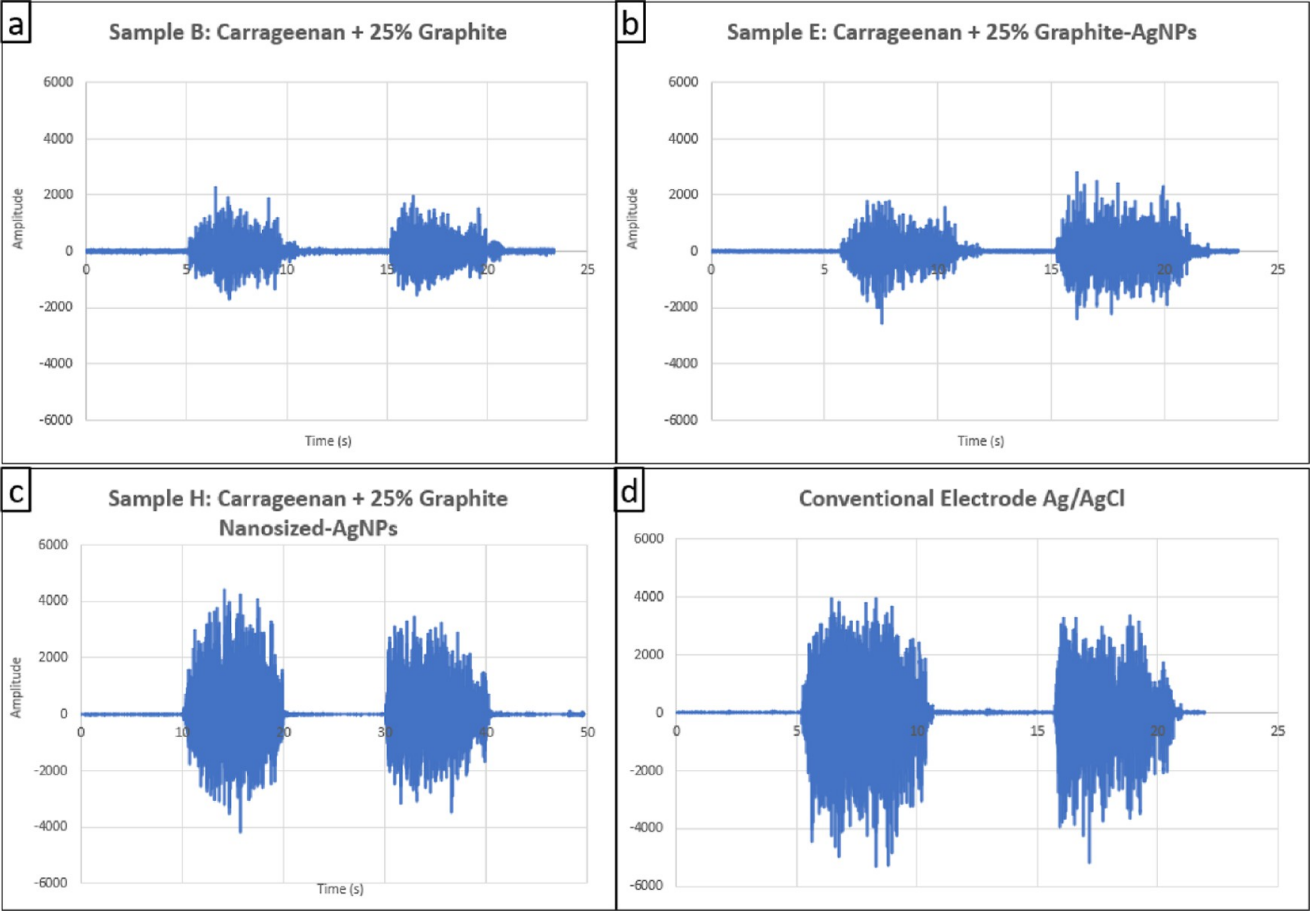
EMG signal measurements of (a) sample B, (b) sample E, (c) sample H, and (d) conventional electrode.

The electrode samples were tested using an EMG under measurement conditions in the hand area. Samples B (25% graphite), E (25% graphite-AgNP), and H (25% graphite nanosized-AgNP) were selected for EMG measurement testing because they have optimal resistivity and conductivity values and can represent other samples as a comparison of different types and contents of conductive material. The EMG signal measurements obtained with the flexible electrode samples are seen in Figure 13, and the results of the SNR are seen in Table 12.

**Table 12:** SNR Values of EMG Signal Measured Using Conventional and Flexible Electrodes

sample	SNR (dB)
B	21.15
E	31.99
G	30.03
H	40.93
I	35.31
conventional	37.45

Sample H has the highest SNR value of 40.93 dB, which is superior to that of the conventional electrode. The addition of the hybrid conductive material graphite-AgNP has an impact on improving the SNR value, as seen in the comparison of EMG signals in Table 12. Sample B has a SNR value of 21.15 dB, while sample E has a SNR value of 31.99 dB, indicating that the increase of the SNR value is approximately 51.3% by AgNP addition. Meanwhile, sample H’s SNR value is better than sample E’s as there is an increase in the SNR value from 31.99 to 40.93 dB, showing that reducing the graphite size can further increase the SNR value by 27.93%. The difference in thickness between samples H and E is only 0.001 mm. However, sample E has a larger graphite size than sample H, ranging from 4500–6500 to 600–700 nm, respectively. Therefore, the size of the hybrid conductive material graphite-AgNP influences the conductivity and SNR of the fabricated flexible bioplastic medical electrode.

The carrageenan-based bioplastic flexible electrodes with the addition of hybrid material graphite nanosized-AgNP with a SNR value of up to 40.93 dB were better than conventional electrode Ag/AgCl, indicating that the bioplastic-based flexible electrode developed in this study has a good performance as a medical electrode for EMG signal measurement.

## Conclusions

The fabrication of flexible medical electrodes using carrageenan-based bioplastic with hybrid graphite-AgNP materials as conductive fillers has been successfully achieved. The performance of the electrodes depends on several parameters, such as the percentage and size of the conductive materials, bioplastic composition, and electrode thickness. Adding a hybrid graphite-AgNP conductive material can enhance the electrical properties but may reduce flexibility. The fabricated thickness of the flexible electrode also affects its electrical and mechanical properties, with thicker electrodes exhibiting lower electrical conductivity and flexibility. The conductive filler’s size reduction also increases the electrical conductivity and flexibility since the filler could spread better and be more evenly distributed. Furthermore, the fabricated flexible electrodes show impedance trends consistent with the conventional Ag/AgCl electrodes, with better performance at lower frequency ranges (20–500 Hz) suitable for EMG signal measurement. The optimal variation of flexible electrodes (sample H) also performs better in EMG measurements based on their higher SNR than conventional electrodes.
